# Genome-wide association study of lung function and clinical implication in heavy smokers

**DOI:** 10.1186/s12881-018-0656-z

**Published:** 2018-08-01

**Authors:** Xingnan Li, Victor E. Ortega, Elizabeth J. Ampleford, R. Graham Barr, Stephanie A. Christenson, Christopher B. Cooper, David Couper, Mark T. Dransfield, Mei Lan K. Han, Nadia N. Hansel, Eric A. Hoffman, Richard E. Kanner, Eric C. Kleerup, Fernando J. Martinez, Robert Paine, Prescott G. Woodruff, Gregory A. Hawkins, Eugene R. Bleecker, Deborah A. Meyers, for the SPIROMICS Research Group, Neil E. Alexis, Wayne H. Anderson, R. Graham Barr, Eugene R. Bleecker, Richard C. Boucher, Russell P. Bowler, Elizabeth E. Carretta, Stephanie A. Christenson, Alejandro P. Comellas, Christopher B. Cooper, David J. Couper, Gerard J. Criner, Ronald G. Crystal, Jeffrey L. Curtis, Claire M. Doerschuk, Mark T. Dransfield, Christine M. Freeman, Mei Lan K. Han, Nadia N. Hansel, Annette T. Hastie, Eric A. Hoffman, Robert J. Kaner, Richard E. Kanner, Eric C. Kleerup, Jerry A. Krishnan, Lisa M. LaVange, Stephen C. Lazarus, Fernando J. Martinez, Deborah A. Meyers, John D. Newell, Elizabeth C. Oelsner, Wanda K. O’Neal, Robert Paine, Nirupama Putcha, Stephen I. Rennard, Donald P. Tashkin, Mary Beth Scholand, J. Michael Wells, Robert A. Wise, Prescott G. Woodruff

**Affiliations:** 1 Division of Genetics, Genomics and Precision Medicine, Department of Medicine, University of Arizona, BioScience Research Lab, Room 253, 1230 N. Cherry Avenue, PO Box 210242, Tucson, AZ 85721 USA; 2 Center for Genomics and Personalized Medicine Research, Wake Forest School of Medicine, Winston-Salem, North Carolina USA; 3 Department of Medicine, Columbia University, New York, NY USA; 4 Division of Pulmonary, Critical Care, Sleep & Allergy, Department of Medicine and Cardiovascular Research Institute, University of California at San Francisco, San Francisco, California USA; 5 Department of Medicine, University of California at Los Angeles, Los Angeles, California USA; 6 Department of Biostatistics, University of North Carolina at Chapel Hill, Chapel Hill, North Carolina USA; 7 Division of Pulmonary, Allergy & Critical Care Medicine, Lung Health Center, University of Alabama at Birmingham, Birmingham, AL USA; 8 Division of Pulmonary & Critical Care, University of Michigan, Ann Arbor, MI USA; 9 Division of Pulmonary and Critical Care Medicine, Johns Hopkins University School of Medicine, Baltimore, MD USA; 10 Department of Radiology, University of Iowa, Iowa City, Iowa USA; 11 Department of Internal Medicine/Pulmonary and Critical Care Medicine, University of Utah, Salt Lake City, UT USA; 12 Department of Medicine, Weill Cornell Medical College of Cornell University, New York, NY USA

**Keywords:** COPD, GWAS, Lung function, rs28929474, SERPINA1, SPIROMICS

## Abstract

**Background:**

The aim of this study is to identify genetic loci associated with post-bronchodilator FEV_1_/FVC and FEV_1_, and develop a multi-gene predictive model for lung function in COPD.

**Methods:**

Genome-wide association study (GWAS) of post-bronchodilator FEV_1_/FVC and FEV_1_ was performed in 1645 non-Hispanic White European descent smokers.

**Results:**

A functional rare variant in *SERPINA1* (rs28929474: Glu342Lys) was significantly associated with post-bronchodilator FEV_1_/FVC (*p* = 1.2 × 10^− 8^) and FEV_1_ (*p* = 2.1 × 10^− 9^). In addition, this variant was associated with COPD (OR = 2.3; *p* = 7.8 × 10^− 4^) and severity (OR = 4.1; *p* = 0.0036). Heterozygous subjects (CT genotype) had significantly lower lung function and higher percentage of COPD and more severe COPD than subjects with the CC genotype. 8.6% of the variance of post-bronchodilator FEV_1_/FVC can be explained by SNPs in 10 genes with age, sex, and pack-years of cigarette smoking (*P* <  2.2 × 10^− 16^).

**Conclusions:**

This study is the first to show genome-wide significant association of rs28929474 in *SERPINA1* with lung function. Of clinical importance, heterozygotes of rs28929474 (4.7% of subjects) have significantly reduced pulmonary function, demonstrating a major impact in smokers. The multi-gene model is significantly associated with CT-based emphysema and clinical outcome measures of severity. Combining genetic information with demographic and environmental factors will further increase the predictive power for assessing reduced lung function and COPD severity.

**Electronic supplementary material:**

The online version of this article (10.1186/s12881-018-0656-z) contains supplementary material, which is available to authorized users.

## Background

Chronic obstructive pulmonary disease (COPD) is a common respiratory disease caused by the interaction of genetic susceptibility with environmental influences, primarily tobacco exposure. COPD is defined as a reduced ratio of post-bronchodilator forced expiratory volume in 1 s (FEV_1_) to forced vital capacity (FVC) (post-bronchodilator FEV1/FVC < 0.70) [1]. COPD severity is measured by the reduction in post-bronchodilator percent predicted FEV_1_, i.e., GOLD stages 1–4 (mild, moderate, severe, and very severe COPD) have post-bronchodilator percent predicted FEV1 ≥ 80%, ≥50%, ≥30%, or < 30%, respectively [1].

Twenty-eight genomic loci associated with baseline FEV_1_/FVC or FEV_1_ have been identified by meta-analyses of genome-wide association studies (GWAS) in general populations of European descent [2–4]. A recent GWAS comparing extremes of high and low baseline FEV_1_ in subjects of European ancestry from the UK Biobank has identified five loci (*KANSL1*, *HLA-DQ*, *NPNT*, *TET2*, and *TSEN54*) in never smokers and *RBM19-TBX5* in heavy smokers [5]. *HHIP*, *FAM13A1*, *CHRNA3*, *RIN3*, *MMP12*, and *TGFB2* have been associated with COPD at genome-wide significant levels [6]. To our knowledge, no GWAS study has been performed on post-bronchodilator FEV_1_/FVC and FEV_1_ in smokers, which defines a diagnosis of COPD and determines COPD severity, respectively.

GWAS of post-bronchodilator FEV_1_/FVC and percent predicted FEV_1_ were performed in non-Hispanic White smokers (*n* = 1645, GOLD stage 0–4, smoking≥20 packs/year) from the NHLBI-sponsored SubPopulations and InteRmediate Outcome Measures In COPD Study (SPIROMICS). In addition to evaluating previously reported loci associated with baseline lung function in general populations, we aimed to identify novel genes associated with abnormal post-bronchodilator lung function in smokers enriched for COPD and develop a model to predict lung function using multiple genes and demographic/environmental factors.

## Methods

### Study subjects

SPIROMICS is a prospective cohort study that enrolled 2981 participants with the goals of identifying new COPD subgroups and intermediate markers of disease progression [7, 8]. SPIROMICS is a well-characterized longitudinal cohort with comprehensive phenotyping including measurements of lung function and quantitative CT scan. Spirometry was performed before and after four inhalations with 90 μg albuterol and 18 μg ipratropium per inhalation according to ATS recommendations. Non-Hispanic White smokers (ever or current smoking≥20 packs/year) with genotyping information available were included in this analysis. Smokers with COPD were defined as smokers (smoking≥20 packs/year) with post-bronchodilator FEV_1_/FVC < 0.7 (GOLD stage 1–4) and ‘healthy’ smoking controls were defined as smokers (smoking≥20 packs/year) with post-bronchodilator FEV_1_/FVC ≥ 0.7 (GOLD stage 0). DNA was isolated using standard protocols, and SNP genotyping performed using Illumina HumanOmniExpressExome BeadChip and BeadStudio (Illumina, Inc., San Diego, CA).

Participants were recruited at each center through physician referral, advertisement in clinical areas or self-referral using the SPIROMICS study website (www.spiromics.com). The research protocol was approved by the institutional review boards of all participating institutions with written informed consent from all participants.

### Statistical analysis

For quality control, subjects were removed if they 1) had genotyping call rates< 95%, 2) were discrepant for genetic sex, 3) failed the check for family relatedness, or 4) were detected as an outlier. After subjects meeting these criteria were excluded, SNPs were removed if 1) call rates< 95%, 2) inconsistent with Hardy-Weinberg Equilibrium (HWE) (*p* < 10^− 6^), or 3) minor allele frequency (MAF) < 0.01.

A linear additive model was used for analysis of pre−/post-bronchodilator FEV_1_/FVC, percent predicted FEV_1_, FVC, and % change in FEV_1_ bronchodilator response using PLINK software (URL: zzz.bwh.harvard.edu/plink/) [9], adjusted for age, sex, current smoking status, pack-years of cigarette smoking, and the first two principal components from the multidimensional scaling analysis of genotypes on the chip. Association analyses of Pre-/Post-bronchodilator FEV_1_ and FVC in ml were performed using linear regression adjusted for sex, age, age^2^, height, height^2^, weight, current smoking status, pack-years of cigarette smoking, and the first two principal components. Association analyses of COPD and COPD severity were performed using logistic regression adjusted for age, sex, current smoking status, pack-years of cigarette smoking, and the first two principal components. *P* values≤5 × 10^− 8^ were considered genome-wide significant. P values ≤0.05 were considered significant for SNP-level evaluation of previously reported candidate SNPs associated with baseline lung function. SNAP software (URL: http://www.broad.mit.edu/mpg/snap/) was used to generate the association plots [10].

Joint analysis of 10 confirmed candidate SNPs was performed, in which eight subjects with homozygous TT genotype of rs28929474 in *SERPINA1* (PiZZ genotype) were not included in joint analysis to avoid bias. Genetic scores were defined by the number of risk alleles presented in these 10 SNPs. A linear model was used for analysis of post-bronchodilator FEV_1_/FVC and percent predicted FEV_1_ with genetic scores in 1632 current or former smokers. Joint analysis of these 10 candidate SNPs was also performed for post-bronchodilator percent predicted FEV_1_ and percentage of subjects with severe COPD (GOLD stage 3–4) in 1077 smokers with COPD.

## Results

### GWAS of post-bronchodilator pulmonary function

After quality control analysis, 1645 non-Hispanic White subjects (1086 subjects with COPD and 559 current and former smokers with preserved lung function [8]) were included in the analysis (Table 1). GWAS of post-bronchodilator FEV_1_/FVC and percent predicted FEV_1_ were performed for 635,970 single nucleotide polymorphisms (SNPs) with MAF ≥ 0.01 in 1645 non-Hispanic White smokers with age, sex, current smoking status, pack-years of cigarette smoking, and the first two principal components as covariates in the linear additive model. Genomic inflation factors are 1.013 and 1.017 for GWAS of post-bronchodilator FEV_1_/FVC and percent predicted FEV_1_, respectively, indicating limited genomic inflation.

**Table 1:** Demographics (Mean ± SD) of Non-Hispanic White Subjects in SPIROMICS

	Cases			Controls			*P* value
	All	Current smokers	Former smokers	All	Current smokers	Former smokers	
n	1086	325	761	559	210	349	NA
Age at enrollment, years	66.2 ± 7.6	62.9 ± 8.1	67.7 ± 6.9	63.6 ± 9.0	58.0 ± 9.1	66.9 ± 7.0	< 0.0001
Female, n (%)	437 (40)	137 (42)	300 (39)	294 (53)	122 (58)	172 (49)	< 0.0001
Body mass index	27.4 ± 5.1	25.7 ± 4.9	28.1 ± 5.0	28.6 ± 5.0	27.5 ± 5.1	29.2 ± 4.8	< 0.0001
Current smokers, n (%)	325 (30)	325 (100)	0 (0)	210 (38)	210 (100)	0 (0)	0.0019
Pack-years of cigarette smoking	55.0 ± 25.7	52.6 ± 24.7	56.0 ± 26.1	46.3 ± 27.3	45.3 ± 31.1	46.9 ± 24.8	< 0.0001
Post-bronchodilator FEV_1_/FVC	0.52 ± 0.13	0.55 ± 0.11	0.50 ± 0.13	0.77 ± 0.05	0.78 ± 0.05	0.77 ± 0.05	< 0.0001
Post-bronchodilator FEV_1_, % predicted	60.1 ± 22.5	63.6 ± 19.7	58.6 ± 23.5	94.4 ± 13.9	93.4 ± 13.2	95.0 ± 14.3	< 0.0001

Subjects with available GWAS genotyping information available at current stage were included; Cases: GOLD stage 1–4; Controls: GOLD stage 0

SNPs in nine genes previously identified for baseline FEV_1_/FVC or FEV_1_ in general populations [2–4], extremes of high and low baseline FEV_1_ [5] or COPD [6] were also associated (*p* < 0.05) with post-bronchodilator FEV_1_/FVC or FEV_1_ (Table 2). SNPs in *RARB*, *HDAC4*, *CHRNA3*, and *RIN3* were associated with post-bronchodilator FEV_1_/FVC and FEV_1_. SNPs in *HHIP*, *AGER*, *FAM13A1*, and *PID1* were only associated with post-bronchodilator FEV_1_/FVC. A SNP in *MMP12* was only associated with post-bronchodilator FEV_1_. The associations were significant at the SNP level with same effect direction as previous findings [2–4, 6].

**Table 2:** Association Results of the Top SNPs (*P* < 10^− 6^) and Candidate Lung Function and COPD SNPs (*P* < 0.05)

SNP	Gene	Chr	Location	Minor (Effect)/Major Allele	Minor AlleleFrequency	Post-bronchodilator FEV_1_/FVC		Post-bronchodilator % predicted FEV_1_	
						β	*P* value	β	*P* value
rs28929474	*SERPINA1*	14	coding	T/C	0.029	−0.087	1.2 × 10^−8^	−13.6	3.5 × 10^− 8^
rs4537555	*HHAT*	1	intron	G/A	0.11	− 0.044	2.1 × 10^− 7^	− 6.3	4.1 × 10^− 6^
rs8079868	*MYH3*	17	3’	C/T	0.12	− 0.034	3.5 × 10^− 5^	−6.7	5.9 × 10^− 7^
rs1980057	*HHIP*	4	5’	T/C	0.37	0.011	0.049	1.4	0.13
rs2070600	*AGER*	6	coding	A/G	0.047	0.026	0.047	3.4	0.10
rs2869967	*FAM13A1*	4	intron	C/T	0.41	−0.014	0.016	−1.4	0.12
rs1435867	*PID1*	2	3’	C/T	0.075	0.021	0.043	1.9	0.26
rs12477314	*HDAC4*	2	3’	T/C	0.21	0.014	0.033	2.3	0.035
rs1529672	*RARB*	3	intron	A/C	0.15	0.026	5.1 × 10^−4^	3.4	5.0 × 10^− 3^
rs12914385	*CHRNA3*	15	intron	T/C	0.43	−0.014	0.014	−2.2	0.014
rs10498635	*RIN3*	14	intron	T/C	0.18	0.021	2.6 × 10^−3^	3.7	1.5 × 10^− 3^
rs615098	*MMP12*	11	3’	A/C	0.18	0.013	0.056	2.4	0.034

Association analyses of Post-bronchodilator % predicted FEV_1_ and FEV_1_/FVC were performed using linear regression adjusted for age, sex, current smoking status, pack-years of cigarette smoking, and the first two principal components

**Fig. 1:**
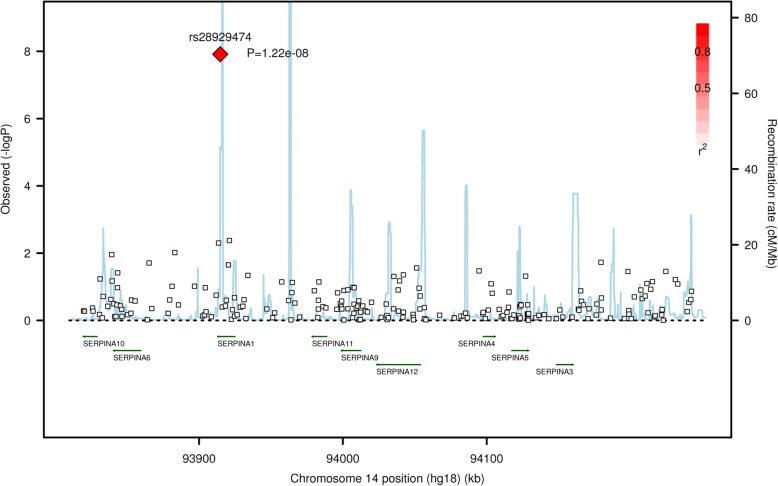
Association plot of *SERPINA1* region with post-bronchodilator FEV_1_/FVC. Negative logarithm-transformed *P* value (*left*) and recombination rate (*right*). Red color scale represents the strength of linkage disequilibrium of SNPs with rs28929474

**Fig. 2:**
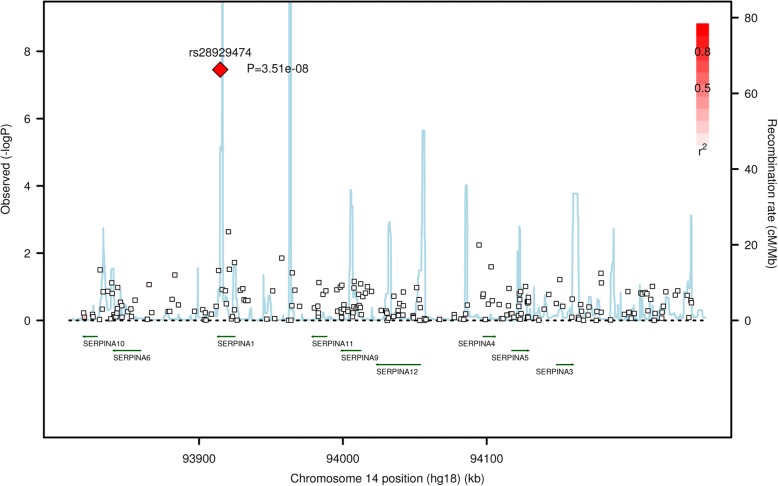
Association plot of *SERPINA1* region with post-bronchodilator percent predicted FEV_1_. Negative logarithm-transformed *P* value (*left*) and recombination rate (*right*). Red color scale represents the strength of linkage disequilibrium of SNPs with rs28929474

rs28929474 (Glu342Lys) in alpha-1 antitrypsin member 1 (*SERPINA1*) was associated with post-bronchodilator FEV_1_/FVC (β = − 0.087, *p* = 1.2 × 10^− 8^) and percent predicted FEV_1_ (β = − 13.6, *p* = 3.5 × 10^− 8^) at a genome-wide significant level (Table 2 and Additional file 1: Tables S1-S2). No other SNPs in the *SERPINA1* region were in strong linkage disequilibrium (LD) with rs28929474 or strongly associated with post-bronchodilator lung function (Figs. 1 and 2). rs4537555 in hedgehog acyltransferase (*HHAT*) and rs8079868 in myosin heavy chain 3 (*MYH3*) were strongly associated with post-bronchodilator FEV_1_/FVC (*p* = 2.1 × 10^− 7^) and percent predicted FEV_1_ (*p* = 5.9 × 10^− 7^), respectively (Table 2 and Additional file 1: Tables S1-S2).

### Association of SERPINA1 with lung function, COPD, and COPD severity

Pre−/post-bronchodilator lung function was stratified by genotypes of rs28929474 (Table 3). rs28929474 was associated in a stepwise fashion with pre−/post-bronchodilator FEV_1_/FVC ratio (0.39, 0.54, and 0.61 for genotype TT, CT, and CC, respectively; p = 1.2 × 10^− 8^). rs28929474 was associated with pre−/post-bronchodilator FEV_1_ (33.5, 61.3, and 72.5 or 1210, 1841, and 2115 ml for genotype TT, CT, and CC, respectively; p = 2.1 × 10^− 9^). Pre−/post-bronchodilator lung function was significantly different between CT and CC or TT and CC genotype groups, however differences between TT and CT genotype groups were not as marked. rs28929474 was associated with post-bronchodilator FEV_1_/FVC (β = − 0.060, *p* = 1.1 × 10^− 5^) and percent predicted FEV_1_ (β = − 8.73; *p* = 2.6 × 10^− 4^) in subjects with COPD (GOLD stage 1–4), but not in subjects without COPD (GOLD stage 0; data not shown). Thus, the association of rs28929474 with lung function was driven by subjects with COPD.

**Table 3:** Association Results of rs28929474 in *SERPINA1* with Lung Function, COPD, and COPD Severity

Phenotype	CC (*n* = 1559)	CT (n = 78)	TT (n = 8)	TT vs. CT vs. CC		CT vs. CC		TT vs. CT		TT vs. CC	
				β or OR	*P* value	β or OR	*P* value	β or OR	*P* value	β or OR	*P* value
Age at enrollment, years	65.4 ± 8.2	64.5 ± 8.1	53.7 ± 3.9	−2.38	0.0029	−0.92	0.33	−10.8	3.6 × 10^−4^	−11.7	5.7 × 10^−5^
Sex (Female vs. Male), n	696 vs. 863	33 vs. 45	2 vs. 6	0.83	0.35	0.91	0.69	0.46	0.35	0.41	0.28
Pack-years of cigarette smoking	52.3 ± 26.6	48.9 ± 26.4	35.2 ± 13.4	−3.97	0.12	−3.10	0.3	−6.62	0.52	−12.3	0.19
Post-bronchodilator FEV_1_/FVC	0.61 ± 0.16	0.54 ± 0.18	0.39 ± 0.09	−0.087	1.2 × 10^−8^	− 0.077	2.3 × 10^− 5^	−0.090	0.21	−0.23	3.2 × 10^− 5^
Pre-bronchodilator FEV_1_/FVC	0.59 ± 0.15	0.52 ± 0.17	0.37 ± 0.09	−0.081	9.9 × 10^− 8^	−0.069	1.7 × 10^− 4^	− 0.086	0.22	−0.22	3.5 × 10^− 5^
Post-bronchodilator % predicted FEV_1_	72.5 ± 25.6	61.3 ± 26.4	33.5 ± 7.89	−13.6	3.5 × 10^− 8^	− 11.4	9.1 × 10^− 5^	−22.5	0.037	−38.1	2.2 × 10^− 5^
Post-bronchodilator FEV_1_, ml	2115 ± 888	1841 ± 860	1210 ± 240	− 439	2.1 × 10^−9^	− 329	1.4 × 10^− 4^	− 1066	1.5 × 10^− 4^	− 1395	1.4 × 10^−7^
Pre-bronchodilator % predicted FEV_1_	65.6 ± 26.1	54.8 ± 26.9	30.2 ± 7.57	−12.9	8.3 × 10^− 7^	−10.9	5.0 × 10^− 4^	−17.5	0.12	−34.7	1.6 × 10^− 4^
Pre-bronchodilator FEV_1_, ml	1916 ± 883	1629 ± 833	1091 ± 240	− 426	1.1 × 10^− 8^	− 339	1.2 × 10^− 4^	− 924	8.1 × 10^− 4^	− 1263	2.9 × 10^− 6^
% change in FEV_1_ (BDR)	13.6 ± 13.5	17.1 ± 18.1	11.6 ± 9.75	2.08	0.13	3.49	0.030	−3.56	0.65	−2.77	0.57
Post-bronchodilator % predicted FVC	90.7 ± 17.7	86.9 ± 17.2	71.5 ± 21.1	−5.23	0.0024	−3.83	0.060	−18.0	0.019	−17.0	0.0070
Post-bronchodilator FVC, ml	3518 ± 1011	3482 ± 1036	3365 ± 1015	− 236	4.6 × 10^− 4^	− 162	0.042	− 661	0.023	− 822	7.1 × 10^− 4^
Pre-bronchodilator % predicted FVC	84.4 ± 19.4	78.9 ± 18.6	68.7 ± 21.7	−6.07	0.0018	−5.55	0.017	−12.6	0.13	−14.2	0.038
Pre-bronchodilator FVC, ml	3274 ± 1032	3145 ± 1015	3240 ± 1071	−277	2.0 × 10^− 4^	− 249	0.0046	− 434	0.16	− 683	0.011
COPD (GOLD stage 2–4 vs. 0), n	803 vs. 539	53 vs. 20	8 vs. 0	2.31	7.8 × 10^−4^	1.91	0.019	NA	NA	NA	NA
COPD severity (GOLD stage 3–4 vs. 1), n	331 vs. 217	32 vs. 5	8 vs. 0	4.08	0.0036	3.79	0.0081	NA	NA	NA	NA

Association analyses of age or sex were performed using linear or logistic regression without adjustment. Association analyses of Pre-/Post-bronchodilator FEV_1_ and FVC in ml were performed using linear regression adjusted for sex, age, age^2^, height, height^2^, weight, current smoking status, pack-years of cigarette smoking, and the first two principal components. Association analyses of Pre-/Post-bronchodilator FEV_1_, FVC, and FEV_1_/FVC, and % change in FEV_1_ were performed using linear regression adjusted for age, sex, current smoking status, pack-years of cigarette smoking, and the first two principal components. Association analyses of COPD and COPD severity were performed using logistic regression adjusted for age, sex, current smoking status, pack-years of cigarette smoking, and the first two principal components

Additional COPD-related phenotypes were analyzed for association with rs28929474 (Table 3). rs28929474 was also associated with COPD status (odds ratio = 2.3, *p* = 7.8 × 10^− 4^) and COPD severity (odds ratio = 4.1, *p* = 0.0036) (Table 3). The percentage of subjects with COPD or severe COPD was significantly higher in subjects with CT genotype than CC genotype. rs28929474 was a less common SNP with minor allele frequency (MAF) of 0.029 in SPIROMICS (Additional file 1: Table S3). Homozygous risk genotype TT was present only in subjects (*n* = 8) with severe COPD (GOLD stage 3–4).

### Prediction of post-bronchodilator pulmonary function

**Fig. 3:**
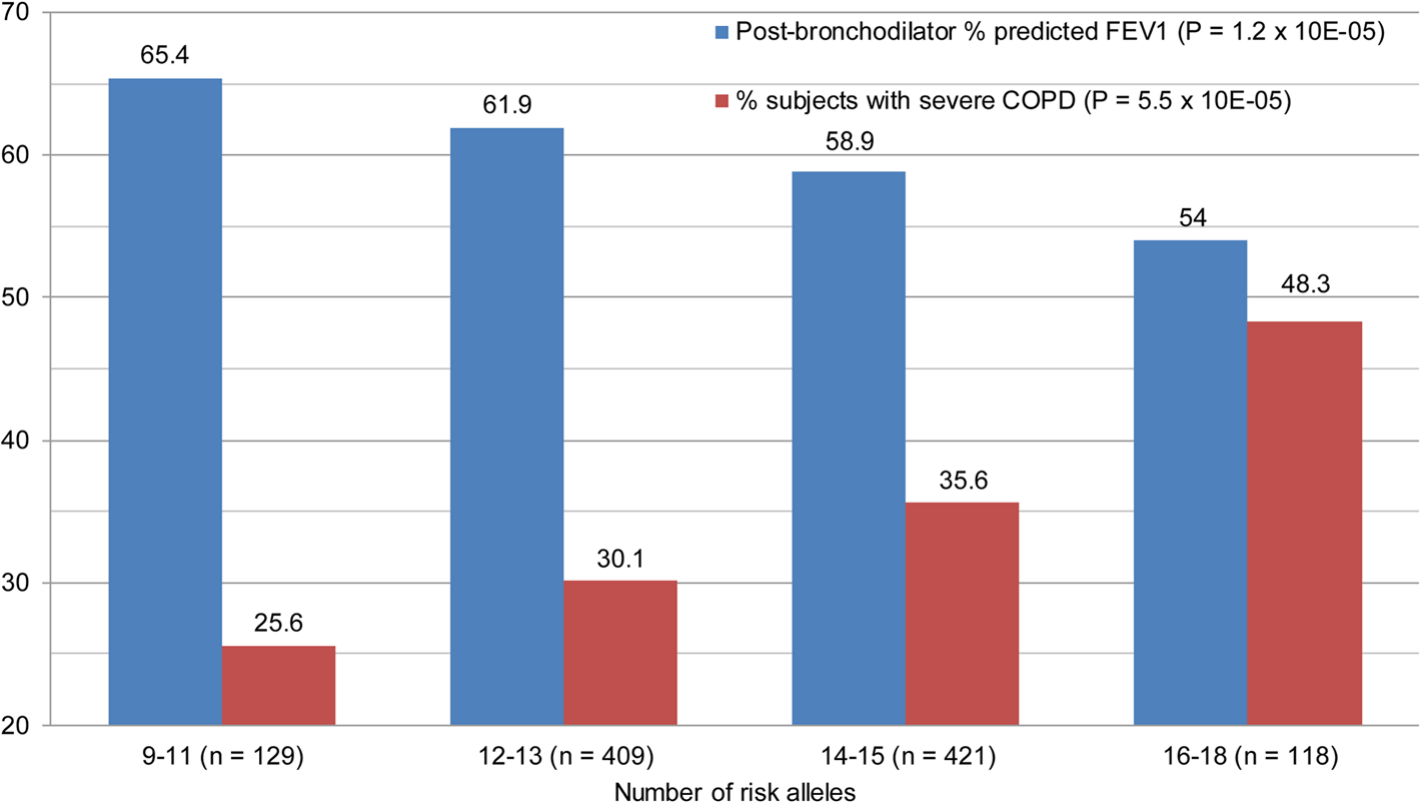
Joint analysis of 10 candidate SNPs in 1077 SPIROMICS non-Hispanic White smokers with COPD. 10 SNPs include rs28929474 in *SERPINA1*, rs1980057 in *HHIP*, rs2869967 in *FAM13A1*, rs2070600 in *AGER*, rs1435867 in *PID1*, rs12477314 in *HDAC4*, rs1529672 in *RARB*, rs12914385 in *CHRNA3*, rs10498635 in *RIN3*, and rs615098 in *MMP12*. Blue bars represent post-bronchodilator percent predicted FEV_1_, and red bars represent percentages of subjects with severe COPD (GOLD stage 3–4)

Joint analysis of the most consistently associated 10 SNPs, based on our analyses and previous findings was performed. Genetic scores (the number of risk alleles) and pack-years of cigarette smoking were significantly associated with post-bronchodilator FEV_1_/FVC and percent predicted FEV_1_ (Table 4). Age at enrollment and sex were significantly associated with post-bronchodilator FEV_1_/FVC but not associated with percent predicted FEV_1_. In 1632 SPIROMICS non-Hispanic White smokers (GOLD stage 0–4), genetic score, age, sex, and pack-years of cigarette smoking explained 3.6, 1.5, 1.9, 3.0%, and together 8.6% of the variance of post-bronchodilator FEV_1_/FVC (Table 4). Genetic score and pack-years of cigarette smoking explained 3.0, 2.9%, and together 5.8% of the variance of post-bronchodilator percent predicted FEV_1_ (Table 4). In 1077 SPIROMICS non-Hispanic White smokers with COPD (GOLD stage 1–4), post-bronchodilator FEV_1_ decreased significantly with the increase in the number of risk alleles, from 65.4 to 54.0 (*p* = 1.2 × 10^− 5^) and the percentage of subjects with severe COPD (GOLD stage 3–4) increased significantly from 25.6 to 48.3% (*p* = 5.5 × 10^− 5^) (Fig. 3).

**Table 4:** Prediction Models for Post-bronchodilator Lung Function

	Post-bronchodilator FEV_1_/FVC			Post-bronchodilator % predicted FEV_1_		
	β	R^2^	*P* value	β	R^2^	*P* value
Genetic Score (8–18)^a^	− 0.018	0.0363	8.6 × 10^− 15^	−2.6	0.0296	2.7 × 10^− 12^
Age at enrollment, years	−0.0024	0.0145	1.1 × 10^− 6^	− 0.042	0.000176	0.59
Sex (Male = 0, Female = 1)	0.044	0.0186	3.1 × 10^− 8^	1.88	0.000132	0.14
Pack-years of cigarette smoking	− 0.0011	0.0304	1.3 × 10^− 12^	−0.017	0.0294	3.2 × 10^− 12^
All	NA	0.0859	< 2.2 × 10^− 16^	NA	0.0583	< 2.2 × 10^− 16^

^a^Genetic scores (the number of risk alleles) of 10 candidate SNPs (rs28929474 in *SERPINA1*, rs1980057 in *HHIP*, rs2869967 in *FAM13A1*, rs2070600 in *AGER*, rs1435867 in *PID1*, rs12477314 in *HDAC4*, rs1529672 in *RARB*, rs12914385 in *CHRNA3*, rs10498635 in *RIN3*, and rs615098 in *MMP12*). 1632 SPIROMICS non-Hispanic White smokers (GOLD stage 0–4) were included. Eight subjects with TT genotype of rs28929474 in *SERPINA1* (PiZZ genotype) were excluded

Joint analysis of the top 10 SNPs associated with post-bronchodilator % predicted FEV_1_ in this study was also performed (Additional file 1: Table S2). In 1634 SPIROMICS non-Hispanic White smokers (GOLD stage 0–4), genetic score, age, sex, and pack-years of cigarette smoking explained 7.5, 1.4, 1.9, 3.1%, and together 12.8% of the variance of post-bronchodilator FEV_1_/FVC (Additional file 1: Table S4). Genetic score and pack-years of cigarette smoking explained 9.9, 3.0%, and together 12.9% of the variance of post-bronchodilator percent predicted FEV_1_ (Additional file 1: Table S4). Increase in the number of risk alleles from 4 to 6 to 11–13 was associated with significant decrease in post-bronchodilator FEV_1_ from 69.4 to 45.6 (*p* <  2.2 × 10^− 16^) and with a significant increase in the percentage of subjects with severe COPD (GOLD stage 3–4) from 21.4 to 57.9% (*p* = 2.2 × 10^− 12^) (Fig. S1).

### Joint analysis of 10 SNPs with emphysema, clinical symptoms, and exacerbation

Joint analysis of 10 candidate SNPs was further performed on quantitative Computed Tomography (CT) evidence of emphysema (TLC % area < − 950 HU) and airtrapping (RV % area < − 856 HU), BODE index, COPD Assessment Test (CAT) score, St. George’s Respiratory Questionnaire (SGRQ) total score, 6-Minute Walk Distance (6MWD), and exacerbations requiring ED visit or hospitalization in last 12 month (Table 5). In general, with the increase of genetic scores, emphysema (*p* < 0.0001) and airtrapping (*p* < 0.0001) increased, BODE index (*p* < 0.0001) and SGRQ total score (*p* = 0.0044) increased, 6MWD (*p* = 0.0086) decreased, and the percentage of subjects with exacerbations (*p* = 0.001) increased. Two extreme genetic score groups (8 to 11 risk alleles vs. 16 to 18 risk alleles) showed statistical and clinical difference for emphysema (5.54 vs. 12.5 of TLC % area < − 950 HU), airtrapping (21.8 vs. 33.9 RV % area < − 856 HU), BODE index (1.15 vs. 2.21), SGRQ total score (30.4 vs. 35.5), 6MWD (416 m vs. 390 m), and percentage of exacerbations (7.5% vs. 14%).

**Table 5:** Joint analysis of 10 SNPs with emphysema, clinical symptoms, and exacerbation

Genetic Score^a^	8–11 (*n* = 228)	12–13 (*n* = 643)	14–15 (*n* = 612)	16–18 (*n* = 149)	*P* valueǂ
CT Evidence of Emphysema (TLC % Area < − 950 HU^b^)	5.54 ± 6.59	7.60 ± 9.84	8.67 ± 10.4	12.5 ± 12.4	< 0.0001
CT Evidence of Airtrapping (RV % Area < − 856 HU^b^)	21.8 ± 18.1	24.0 ± 20.6	27.6 ± 21.0	33.9 ± 23.2	< 0.0001
BODE Index	1.15 ± 1.62	1.33 ± 1.75	1.61 ± 1.98	2.21 ± 2.41	< 0.0001
COPD Assessment Test (CAT)	12.7 ± 8.0	13.0 ± 8.0	14.0 ± 8.2	13.5 ± 7.9	0.07
St. George’s Respiratory Questionnaire (SGRQ) Total Score	30.4 ± 19.7	30.3 ± 19.6	32.9 ± 20.0	35.5 ± 20.6	0.0044
6-Minute Walk Distance (6MWD, meters)	416 ± 114	415 ± 111	418 ± 124	390 ± 126	0.0086
Exacerbations requiring ED Visit or Hospitalization in last 12 months (% Yes)	7.5%	6.5%	11%	14%	0.001

^a^Genetic scores (the number of risk alleles) of 10 candidate SNPs (rs28929474 in *SERPINA1*, rs1980057 in *HHIP*, rs2869967 in *FAM13A1*, rs2070600 in *AGER*, rs1435867 in *PID1*, rs12477314 in *HDAC4*, rs1529672 in *RARB*, rs12914385 in *CHRNA3*, rs10498635 in *RIN3*, and rs615098 in *MMP12*). 1632 SPIROMICS non-Hispanic White smokers (GOLD stage 0–4) were included. Eight subjects with TT genotype of rs28929474 in *SERPINA1* (PiZZ genotype) were excluded. ^b^ CT scan-based measures of emphysema (− 950 Hounsfield Units or less [%Bilateral Lung Area ≤ − 950]) and airtrapping (− 856 Hounsfield Units or less [%Bilateral Lung Area ≤ − 856]) measures log-transformed for analysis and adjusted by study site, age, sex, height, BMI, and pack-year smoking history. ǂGeneralized linear model was used with adjusted of age, sex, current smoking status, and pack-years of cigarette smoking. In generalized linear models, CT evidence of emphysema and airtrapping were natural logarithm transformed; 6MWD was logarithm (base 10) transformed

## Discussion

In this study, we performed GWAS of post-bronchodilator FEV_1_/FVC and percent predicted FEV_1_, and identified rs28929474 in *SERPINA1*. In 1963, Laurell and Eriksson identified the connection between alpha 1-antitrypsin (A1AT) deficiency and degenerative pulmonary disease [11]. The *SERPINA1* gene on chr14q32 encodes A1AT protein. The most common variant of *SERPINA1* causing A1AT deficiency is the Z allele (rs28929474: Glu342Lys), which is a missense mutation of glutamic acid to lysine at position 342 of A1AT protein. The homozygous TT genotype of rs28929474 (PiZZ genotype) is consistently associated with emphysema, decreased lung function, and COPD [12, 13].

Previous GWAS of COPD, emphysema, and lung function did not identify rs28929474 in *SERPINA1* [2–6, 14]. There are several potential reasons for missing this association. rs28929474 is relatively rare in the general population, for example, approximately 2 and 0.01% of the population in the United States are heterozygous or homozygous for the T allele, respectively [15]. The largest meta-analyses of GWAS of baseline lung function in general populations of European descent [2–5] have included tens of thousands subjects, however very few subjects may have been homozygous for the T allele and more importantly these studies did not ascertain subjects with a significant history of cigarette smoking, a necessary environmental exposure. Thus, these studies in general populations have limited power to identify the association between rs28929474 and lung function. In this study, we performed GWAS of post-bronchodilator lung function in heavy smokers enriched for COPD. As expected the number of subjects with homozygous TT genotype was rare (*n* = 8 in 1645 or 0.49%) but the heterozygous CT genotype was more common (*n* = 78 or 4.74%). In addition, rs28929474 is not included in the previously designed GWAS chips nor are there other SNPs in strong LD (r^2^ > 0.5) with rs28929474, preventing the identification of association with COPD and emphysema [6, 14]. The Illumina OmniExpressExome BeadChip used in this study includes exonic markers identified from exome and whole genome sequencing projects. rs28919474 (exm1124179) was directly genotyped. This study found rs28929474 in *SERPINA1* to be associated with pre- and post-bronchodilator FEV_1_/FVC and FEV_1_ at a genome-wide significant level (Table 3).

Although the function of homozygous TT has been known for a long while, the effect of heterozygous CT is more controversial and has been questioned in candidate-gene studies in the past [16–18]. For example, in a general population (*n* = 4600), baseline FEV_1_/FVC and FEV_1_ were not significantly different between PiMM and PiMZ [17]. In a case-control study (834 COPD cases and 835 controls), post-bronchodilator FEV_1_/FVC and FEV_1_ were not significantly different between PiMM and PiMZ [16]. In a small study composed of mainly healthy subjects, post-bronchodilator FEV_1_/FVC (0.77 or 0.71 for PiMM or PiMZ) and percent predicted FEV_1_ (96.4 or 84.6 for PiMM or PiMZ) were significantly different in ever-smokers but not in never-smokers [18]. In a recent candidate-gene study (5518 non-Hispanic Whites and 2753 African Americans with ≥10 pack-years of smoking), subjects with PiMZ had significant lower lung function than subjects with PiMM in both Whites and African Americans [19]. In the current study, subjects with CT genotype had intermediate values for lung function between subjects with TT and CC genotype (Table 3). Subjects with CT genotypes had significantly lower post-bronchodilator FEV_1_/FVC and percent predicted FEV_1_, and higher percentage of COPD and more severe COPD than subjects with CC genotype. Thus, *SERPINA1* CT heterozygosity has important functional effects on COPD and lung function. All subjects included in our study had a history of tobacco smoking with at least a 20-pack-years. Association results were unaffected by the number of pack-years of cigarette smoking in our study. Compared with results from COPDGene study [19], this study included heavier smokers, and thus had lower lung function. More importantly, this study is a hypothesis-free GWAS study, which identified association of rs28929474 with lung function at genome-wide significant level for the first time. More than a hundred common and rare variants exist in the *SERPINA1* gene. Thun et al. have identified synthetic association between common variants in *SERPINA1* and serum A1AT levels, suggesting A1AT levels are causally determined by rare variants such as Z allele and S allele (rs17580) [20]. Cho et al. have identified rs45505795 in *SERPINA10* with MAF of 0.04 (not in strong LD with rs28929474: r^2^ = 0.295) associated with emphysema [14]. We found no SNP other than rs28929474 in *SERPINA1* region to be strongly associated with lung function (Figs. 1 and 2).

To develop a multi-gene predictive model for lung function, genes associated with lung function and COPD in previous published studies were evaluated. We identified the association of *HHIP*, *FAM13A1*, *AGER*, *PID1*, *HDAC4*, *RARB*, *CHRNA3*, *RIN3*, and *MMP12* with post-bronchodilator lung function at the SNP level (Table 2). In a previous study, we have showed that *HHIP*, *FAM13A1*, *AGER* and *RARB* associated with pre-bronchodilator lung function in subjects with asthma [21]. The lung expression quantitative trait locus (eQTL) analysis has identified *cis*-eQTL SNPs in *HHIP*, *FAM13A1*, and *AGER* [22]. All the evidence indicates rs1980057 in *HHIP*, rs2869967 in *FAM13A1*, and rs2070600 in *AGER* are functionally relevant SNPs important for lung function in the general population and in subjects with COPD or asthma. rs4537555 in *HHAT* was strongly associated with post-bronchodilator FEV_1_/FVC (Table 2). *HHAT* is a hedgehog acyltransferase which catalyzes N-terminal palmitoylation of sonic hedgehog (SHH). Hedgehog interacting protein (*HHIP*) and patched homolog 1 (*PTCH1*) are the other two genes involved in hedgehog signaling pathway and associated with lung function [2–4, 21], indicating the importance of this pathway in lung development and function. Independent replication and functional study of *HHAT* are warranted.

Since each of these variants alone had smaller effects, we performed a joint analysis of 10 confirmed candidate SNPs. This analysis explained 3.63 and 2.96% variance of post-bronchodilator FEV_1_/FVC and percent predicted FEV_1_, respectively (Table 4). In contrast, pack-years of cigarette smoking explained 3.04 and 2.94% variance of post-bronchodilator FEV_1_/FVC and percent predicted FEV_1_. A genetic score using these 10 candidate SNPs, age, sex, and pack-years of cigarette smoking together explained 8.59 and 5.83% variance of post-bronchodilator FEV_1_/FVC and percent predicted FEV_1_. In addition, joint analysis of 10 confirmed candidate SNPs (with Z allele homozygotes removed) was performed on CT evidence of emphysema and airtrapping, BODE index, COPD, CAT score, SGRQ total score, 6MWD, and exacerbations (Table 5) in all heavy smokers (Gold stage 0–4). Statistical and clinical significant difference was shown between two extreme genetic score groups (8–11 vs. 16–18) for emphysema, airtrapping, BODE index, SGRQ total score, 6MWD, and exacerbations, indicating the potential usefulness of genetic information to distinguish clinical subgroups of heavy smokers. It will be important to evaluate the power of this model to predict decline in lung function and progression of COPD severity longitudinally in clinical settings.

In summary, rs28929474 in *SERPINA1* is clearly associated with post-bronchodilator FEV_1_/FVC and FEV_1_ among heavy smokers. This study is the first to show genome-wide significant association of rs28929474 with lung function. In addition, rs28929474 is associated with COPD and COPD severity. While well-established rare ZZ homozygotes have severe COPD and emphysema, this study establishes that more common heterozygotes (4.7% of subjects) at this locus lead to pulmonary abnormality in smokers and COPD. Thus, in future clinical studies, this largely ignored heterozygotes group should be carefully examined. A joint genetic model combined with environmental factors is associated with reduced lung function, emphysema, exacerbation, and clinical symptoms. The models should be tested in other populations as well as longitudinally to evaluate potential value of predicting COPD progression and severity.

## Additional file

Additional file 1: **Table S1.** Association Results of the Top SNPs (*P* < 10^− 4^) with Post-bronchodilator FEV_1_/FVC. **Table S2.** Association Results of the Top SNPs (*P* < 10^− 4^) with Post-bronchodilator % Predicted FEV_1_.**Table S3.** Genotype Frequency of rs28929474 in *SERPINA1* Stratified by GOLD Stages. **Table S4.** Prediction Models for Post-bronchodilator Lung Function Using Top 10 SNPs for Post-bronchodilator % Predicted FEV_1_.**Figure S1.** Joint analysis of the top10 SNPs for post-bronchodilator % predicted FEV_1_ in 1075 SPIROMICS non-Hispanic White smokers with COPD. (DOCX 141 kb)

## Abbreviations

COPD: Chronic obstructive pulmonary disease
FEV_1_: Forced expiratory volume in 1 s
GWAS: Genome-wide association studies
SPIROMICS: The SubPopulations and InteRmediate Outcome Measures In COPD Study
