# Supplementary material for: Genome-wide association study of lung function and clinical implication in heavy smokers
Source: BMC Med Genet. 2018 Aug 1;19:134. doi: 10.1186/s12881-018-0656-z (PMC6090900; doi:10.1186/s12881-018-0656-z)

**Online Supplemental Material**

**Genome-wide association study of lung function and clinical implication in heavy smokers**

Xingnan Li, Victor E. Ortega, Elizabeth J. Ampleford, R Graham Barr, Stephanie A. Christenson, Christopher B. Cooper, David Couper, Mark T. Dransfield, MeiLan K. Han, Nadia N. Hansel, Eric A. Hoffman, Richard E. Kanner, Eric C. Kleerup, Fernando J. Martinez, Robert Paine III, Prescott G. Woodruff , Gregory A. Hawkins, Eugene R. Bleecker, Deborah A. Meyers, for the SPIROMICS Research Group

**Correspondence:**

Xingnan Li, PhD, MS, Division of Genetics, Genomics and Precision Medicine, Department of Medicine, University of Arizona. UA Cancer Center, Room 2964, PO Box 245099, 1515 N. Campbell Avenue, Tucson, AZ 85724, USA. Email: [lixingnan1@deptofmed.arizona.edu](mailto:lixingnan1@deptofmed.arizona.edu)

**Table S1.** Association Results of the Top SNPs (P < 10^-4^) with Post-bronchodilator FEV_1_/FVC

| SNP | Chr | Gene | Minor  (Effect)  Allele | Post-bronchodilator  FEV_1_/FVC | | Post-bronchodilator  % predicted FEV_1_ | | COPD  (GOLD stage 2-4 vs. 0) | | COPD severity  (GOLD stage 3-4 vs. 1) | |
| --- | --- | --- | --- | --- | --- | --- | --- | --- | --- | --- | --- |
|  |  |  |  | β | P value | β | P value | OR | P value | OR | P value |
| rs28929474 | 14 | *SERPINA1* | T | -0.08749 | 1.22E-08 | -13.62 | 3.51E-08 | 2.312 | 7.82E-04 | 4.077 | 3.57E-03 |
| rs4537555 | 1 | *HHAT* | G | -0.04418 | 2.08E-07 | -6.306 | 4.12E-06 | 1.272 | 5.23E-02 | 1.593 | 1.81E-02 |
| rs10503023 | 18 | *SEC11L3* | A | -0.03109 | 1.34E-06 | -4.581 | 9.57E-06 | 1.379 | 7.54E-04 | 1.392 | 2.67E-02 |
| rs2210865 | 9 | *DMRTA1* | G | -0.02653 | 1.81E-06 | -3.454 | 1.13E-04 | 1.302 | 1.22E-03 | 1.202 | 1.54E-01 |
| rs12551699 | 9 | *DMRTA1* | C | -0.02633 | 2.12E-06 | -3.4 | 1.43E-04 | 1.297 | 1.43E-03 | 1.202 | 1.54E-01 |
| rs17016283 | 1 | *HHAT* | A | -0.0435 | 2.23E-06 | -6.777 | 4.59E-06 | 1.427 | 8.82E-03 | 1.824 | 5.06E-03 |
| rs862225 | 16 | *JPH3* | A | 0.04879 | 2.63E-06 | 6.611 | 7.69E-05 | 0.5239 | 1.73E-05 | 0.6117 | 5.23E-02 |
| rs17167368 | 7 | *ETV1* | G | 0.101 | 2.92E-06 | 15.55 | 7.66E-06 | 0.2267 | 2.32E-05 | 0.4482 | 2.90E-01 |
| rs17695493 | 9 | *DMRTA1* | C | -0.02586 | 3.59E-06 | -3.097 | 5.69E-04 | 1.276 | 2.72E-03 | 1.184 | 2.06E-01 |
| rs4133767 | 9 | *DMRTA1* | G | -0.02598 | 4.03E-06 | -3.205 | 4.12E-04 | 1.323 | 7.04E-04 | 1.1 | 4.62E-01 |
| rs6753618 | 2 | *GPR39* | A | 0.02683 | 6.13E-06 | 4.316 | 6.10E-06 | 0.7194 | 1.25E-04 | 0.6419 | 2.01E-03 |
| rs7514691 | 1 | *HHAT* | G | -0.02465 | 6.58E-06 | -3.666 | 3.09E-05 | 1.226 | 1.10E-02 | 1.489 | 2.47E-03 |
| rs926579 | 1 | *HHAT* | A | -0.02512 | 7.26E-06 | -3.243 | 3.22E-04 | 1.277 | 2.72E-03 | 1.305 | 4.21E-02 |
| rs360178 | 14 | *RPL10L* | T | 0.03053 | 8.82E-06 | 4.3 | 1.02E-04 | 0.6874 | 1.48E-04 | 0.7888 | 1.51E-01 |
| rs1375290 | 9 | *DMRTA1* | C | -0.02536 | 9.88E-06 | -3.337 | 3.03E-04 | 1.307 | 1.52E-03 | 1.147 | 2.91E-01 |
| rs2357587 | 1 | *HHAT* | C | 0.02397 | 1.10E-05 | 3.534 | 5.63E-05 | 0.7216 | 4.19E-05 | 0.7739 | 4.61E-02 |
| rs12161880 | 14 | *RPL10L* | A | 0.02984 | 1.38E-05 | 4.294 | 1.01E-04 | 0.6944 | 2.13E-04 | 0.7784 | 1.29E-01 |
| rs7156844 | 14 | *RPL10L* | C | 0.02984 | 1.38E-05 | 4.294 | 1.01E-04 | 0.6944 | 2.13E-04 | 0.7784 | 1.29E-01 |
| exm703363 | 8 | *BHLHB5* | G | -0.02679 | 1.48E-05 | -3.63 | 2.65E-04 | 1.357 | 7.34E-04 | 1.556 | 2.91E-03 |
| rs1010777 | 9 | *ANP32B* | C | 0.02346 | 1.59E-05 | 3.114 | 3.74E-04 | 0.7663 | 8.00E-04 | 0.8967 | 3.90E-01 |
| rs12021819 | 1 | *PLA2G2A* | C | -0.03038 | 1.65E-05 | -4.143 | 2.63E-04 | 1.297 | 1.30E-02 | 1.19 | 2.77E-01 |
| rs902391 | 6 | *FRMD1* | G | 0.02821 | 1.70E-05 | 3.582 | 6.91E-04 | 0.7708 | 5.23E-03 | 0.8408 | 2.84E-01 |
| rs12748886 | 1 | *HHAT* | G | 0.02381 | 1.78E-05 | 3.783 | 2.25E-05 | 0.8021 | 6.52E-03 | 0.6827 | 4.29E-03 |
| rs12553349 | 9 | *DMRTA1* | C | -0.02371 | 2.10E-05 | -2.811 | 1.74E-03 | 1.258 | 4.70E-03 | 1.152 | 2.85E-01 |
| rs1888924 | 9 | *DAPK1* | G | 0.03926 | 2.30E-05 | 6.495 | 1.33E-05 | 0.6969 | 6.46E-03 | 0.5023 | 2.27E-03 |
| rs1045643 | 16 | *UBN1* | T | -0.03336 | 2.66E-05 | -4.92 | 1.18E-04 | 1.555 | 2.28E-04 | 1.269 | 1.87E-01 |
| rs6014468 | 20 | *CBLN4* | A | 0.02841 | 2.75E-05 | 3.446 | 1.58E-03 | 0.7118 | 5.06E-04 | 0.876 | 4.18E-01 |
| rs1891792 | 10 | *TCERG1L* | G | -0.02292 | 3.00E-05 | -3.354 | 1.48E-04 | 1.298 | 1.04E-03 | 1.27 | 6.17E-02 |
| rs2280870 | 8 | *BHLHB5* | C | -0.02583 | 3.07E-05 | -3.5 | 4.48E-04 | 1.327 | 1.78E-03 | 1.524 | 4.55E-03 |
| rs7099487 | 10 | *RPS24* | T | -0.02676 | 3.16E-05 | -3.877 | 1.79E-04 | 1.331 | 2.37E-03 | 1.572 | 3.81E-03 |
| rs6567036 | 18 | *ZNF532* | C | -0.0295 | 3.23E-05 | -3.441 | 2.60E-03 | 1.455 | 4.45E-04 | 0.9977 | 9.89E-01 |
| rs7649058 | 3 | *CPNE4* | C | -0.02484 | 3.26E-05 | -3.607 | 1.78E-04 | 1.307 | 2.02E-03 | 1.6 | 1.38E-03 |
| rs4601592 | 1 | *HHAT* | T | -0.02275 | 3.49E-05 | -3.239 | 2.51E-04 | 1.318 | 5.75E-04 | 1.229 | 1.16E-01 |
| rs8079868 | 17 | *MYH3* | C | -0.03441 | 3.50E-05 | -6.669 | 5.93E-07 | 1.676 | 4.48E-05 | 1.501 | 3.80E-02 |
| rs1320903 | 3 | *CPNE4* | T | -0.02463 | 3.92E-05 | -3.532 | 2.46E-04 | 1.305 | 2.24E-03 | 1.593 | 1.52E-03 |
| rs4737675 | 8 | *BHLHB5* | T | -0.02715 | 4.02E-05 | -3.788 | 3.72E-04 | 1.365 | 1.37E-03 | 1.517 | 7.63E-03 |
| rs1923786 | 9 | *FLJ45537* | A | 0.04795 | 4.37E-05 | 7.374 | 9.34E-05 | 0.5707 | 1.04E-03 | 0.4945 | 1.62E-02 |
| rs1266831 | 6 | *IL17F* | C | 0.02457 | 4.77E-05 | 3.206 | 9.78E-04 | 0.7885 | 6.15E-03 | 0.8151 | 1.54E-01 |
| rs7200819 | 16 | *PPL* | G | -0.02974 | 5.07E-05 | -4.314 | 2.56E-04 | 1.558 | 7.30E-05 | 1.236 | 2.05E-01 |
| rs863035 | 1 | *IGSF4B* | T | -0.02655 | 5.17E-05 | -3.59 | 6.71E-04 | 1.421 | 3.47E-04 | 1.144 | 3.73E-01 |
| rs3000860 | 1 | *LOC12676* | T | -0.03307 | 5.18E-05 | -5.532 | 2.55E-05 | 1.443 | 2.60E-03 | 1.709 | 7.78E-03 |
| rs4831399 | 8 | *DLC1* | C | -0.02385 | 5.33E-05 | -3.879 | 4.39E-05 | 1.433 | 4.36E-05 | 1.242 | 1.18E-01 |
| rs7836885 | 8 | *TRPA1* | G | 0.02583 | 5.35E-05 | 3.692 | 3.33E-04 | 0.7305 | 7.17E-04 | 0.7886 | 1.19E-01 |
| rs2567588 | 5 | *MARCH6* | G | -0.03571 | 5.38E-05 | -5.648 | 7.14E-05 | 1.539 | 1.22E-03 | 1.591 | 2.31E-02 |
| rs10863837 | 1 | *HHAT* | A | 0.02251 | 5.62E-05 | 3.285 | 2.58E-04 | 0.7769 | 1.98E-03 | 0.6677 | 2.52E-03 |
| rs9295041 | 6 | *FRMD1* | A | 0.02874 | 6.03E-05 | 3.987 | 5.43E-04 | 0.7481 | 4.53E-03 | 0.7836 | 1.65E-01 |
| rs17016349 | 1 | *HHAT* | A | -0.0261 | 6.10E-05 | -4.117 | 8.46E-05 | 1.174 | 8.77E-02 | 1.437 | 1.80E-02 |
| rs9900684 | 17 | *TAOK1* | T | -0.02727 | 6.12E-05 | -3.32 | 2.44E-03 | 1.246 | 2.70E-02 | 1.254 | 1.48E-01 |
| rs2826587 | 21 | *NCAM2* | G | 0.02756 | 6.16E-05 | 2.927 | 8.23E-03 | 0.8032 | 2.64E-02 | 0.6079 | 2.58E-03 |
| rs243957 | 4 | *ENPEP* | T | 0.02381 | 6.29E-05 | 3.762 | 8.46E-05 | 0.7376 | 3.71E-04 | 0.8329 | 1.91E-01 |
| rs3176875 | 1 | *VCAM1* | A | 0.07501 | 6.63E-05 | 8.5 | 5.00E-03 | 0.3459 | 7.69E-05 | 0.7939 | 7.07E-01 |
| rs2962318 | 5 | *ROPN1L* | G | -0.03597 | 6.74E-05 | -6.184 | 2.02E-05 | 1.619 | 4.74E-04 | 1.552 | 3.67E-02 |
| rs2302976 | 5 | *PIK3R1* | G | -0.02688 | 6.80E-05 | -3.768 | 5.21E-04 | 1.406 | 8.10E-04 | 1.23 | 1.82E-01 |
| rs11793406 | 9 | *DMRTA1* | G | -0.0223 | 6.85E-05 | -2.798 | 1.92E-03 | 1.247 | 6.94E-03 | 1.093 | 4.96E-01 |
| rs6719286 | 2 | *POU3F3* | A | 0.02253 | 6.97E-05 | 3.423 | 1.72E-04 | 0.7729 | 1.63E-03 | 0.908 | 4.67E-01 |
| rs507662 | 6 | *SENP6* | T | -0.07096 | 7.05E-05 | -10.39 | 2.96E-04 | 1.702 | 4.71E-02 | 2.098 | 9.05E-02 |
| rs4588902 | 8 | *TRPA1* | A | 0.02503 | 7.15E-05 | 3.254 | 1.34E-03 | 0.7692 | 3.84E-03 | 0.8427 | 2.56E-01 |
| rs12825792 | 12 | *CCDC59* | C | -0.02492 | 7.25E-05 | -3.652 | 3.01E-04 | 1.287 | 6.36E-03 | 1.258 | 1.11E-01 |
| rs11746935 | 5 | *FBN2* | A | 0.02315 | 7.28E-05 | 3.02 | 1.32E-03 | 0.7978 | 7.03E-03 | 0.7619 | 5.43E-02 |
| exm91581 | 1 | *ANKRD35* | G | -0.1021 | 7.31E-05 | -14.62 | 4.21E-04 | 5.151 | 2.35E-03 | 2.525 | 1.71E-01 |
| rs7837524 | 8 | *ST3GAL1* | T | 0.03738 | 7.54E-05 | 5.458 | 3.27E-04 | 0.6375 | 8.49E-04 | 0.6178 | 3.53E-02 |
| rs10177527 | 2 | *GPR39* | C | 0.02179 | 7.67E-05 | 3.491 | 8.15E-05 | 0.7585 | 5.41E-04 | 0.7658 | 3.82E-02 |
| rs11689323 | 2 | *GPR39* | G | 0.02179 | 7.67E-05 | 3.491 | 8.15E-05 | 0.7585 | 5.41E-04 | 0.7658 | 3.82E-02 |
| rs556436 | 11 | *MGC13125* | G | -0.02417 | 7.71E-05 | -3.455 | 4.44E-04 | 1.234 | 1.93E-02 | 1.396 | 1.49E-02 |
| rs2153903 | 1 | *SLC45A3* | A | -0.0229 | 7.71E-05 | -3.018 | 1.21E-03 | 1.252 | 7.91E-03 | 1.215 | 1.50E-01 |
| rs2936582 | 5 | *ROPN1L* | A | -0.03577 | 7.78E-05 | -6.235 | 1.83E-05 | 1.63 | 4.17E-04 | 1.575 | 3.24E-02 |
| rs17447545 | 20 | *PLTP* | G | 0.02748 | 7.87E-05 | 3.578 | 1.40E-03 | 0.7301 | 1.47E-03 | 1.045 | 7.92E-01 |
| rs1923783 | 9 | *FLJ45537* | A | 0.04668 | 7.91E-05 | 7.239 | 1.42E-04 | 0.5652 | 9.20E-04 | 0.516 | 2.49E-02 |
| rs4778591 | 15 | *ARNT2* | A | 0.03067 | 8.05E-05 | 3.376 | 7.03E-03 | 0.7139 | 2.11E-03 | 0.794 | 2.40E-01 |
| rs17041090 | 2 | *NRXN1* | T | 0.04446 | 8.09E-05 | 6.802 | 1.77E-04 | 0.4849 | 1.05E-05 | 0.6818 | 1.59E-01 |
| rs11895798 | 2 | *POU3F3* | T | 0.02993 | 8.11E-05 | 3.488 | 4.33E-03 | 0.6885 | 6.49E-04 | 0.7058 | 6.10E-02 |
| rs2589668 | 5 | *MARCH6* | C | -0.03492 | 8.27E-05 | -5.398 | 1.55E-04 | 1.545 | 1.15E-03 | 1.547 | 3.29E-02 |
| rs733659 | 2 | *RBMS1* | T | -0.02685 | 8.38E-05 | -3.542 | 1.27E-03 | 1.242 | 3.00E-02 | 1.557 | 6.03E-03 |
| rs9419364 | 10 | *C10orf39* | C | -0.02832 | 8.41E-05 | -3.739 | 1.26E-03 | 1.33 | 7.05E-03 | 1.322 | 9.92E-02 |
| rs11556024 | 11 | *MGC13125* | T | -0.1038 | 9.07E-05 | -16.44 | 1.16E-04 | 3.649 | 4.98E-03 | 4.88 | 4.99E-02 |
| rs7814393 | 8 | *BHLHB5* | C | -0.02503 | 9.07E-05 | -3.261 | 1.53E-03 | 1.378 | 6.60E-04 | 1.507 | 6.98E-03 |
| rs4422885 | 9 | *C9orf102* | A | 0.03924 | 9.17E-05 | 5.304 | 1.02E-03 | 0.6884 | 8.72E-03 | 0.726 | 1.93E-01 |
| rs13266548 | 8 | *TRPA1* | C | 0.02465 | 9.21E-05 | 3.2 | 1.61E-03 | 0.7706 | 4.10E-03 | 0.8467 | 2.70E-01 |
| rs4845025 | 1 | *HHAT* | G | -0.02159 | 9.21E-05 | -2.93 | 9.76E-04 | 1.24 | 7.49E-03 | 1.221 | 1.20E-01 |
| rs13155132 | 5 | *MARCH6* | T | -0.03479 | 9.24E-05 | -5.45 | 1.40E-04 | 1.523 | 1.66E-03 | 1.547 | 3.29E-02 |
| rs7897877 | 10 | *C10orf39* | T | -0.02818 | 9.31E-05 | -3.73 | 1.31E-03 | 1.342 | 5.48E-03 | 1.328 | 9.37E-02 |
| rs7679 | 20 | *C20orf67* | C | 0.02725 | 9.37E-05 | 3.377 | 2.63E-03 | 0.7459 | 3.08E-03 | 1.069 | 6.92E-01 |
| rs26740 | 5 | *MYO10* | T | -0.09155 | 9.49E-05 | -10.75 | 4.43E-03 | 2.004 | 6.29E-02 | 2.614 | 6.66E-02 |
| rs1519627 | 2 | *POU3F3* | A | 0.0296 | 9.53E-05 | 3.405 | 5.32E-03 | 0.6934 | 8.18E-04 | 0.713 | 6.85E-02 |
| rs12656176 | 5 | *PIK3R1* | A | -0.02641 | 9.65E-05 | -3.824 | 4.50E-04 | 1.412 | 7.44E-04 | 1.216 | 2.07E-01 |

Association analyses of Post-bronchodilator % predicted FEV_1_ and FEV_1_/FVC were performed using linear regression adjusted for age, sex, current smoking status, pack-years of cigarette smoking, and the first two principal components. Association analyses of COPD and COPD severity were performed using logistic regression adjusted for age, sex, current smoking status, pack-years of cigarette smoking, and the first two principal components.

**Table S2.** Association Results of the Top SNPs (P < 10^-4^) with Post-bronchodilator % Predicted FEV_1_

| SNP | Chr | Gene | Minor  (Effect)  Allele | Post-bronchodilator  % predicted FEV_1_ | | Post-bronchodilator  FEV_1_/FVC | | COPD  (GOLD stage 2-4 vs. 0) | | COPD severity  (GOLD stage 3-4 vs. 1) | |
| --- | --- | --- | --- | --- | --- | --- | --- | --- | --- | --- | --- |
|  |  |  |  | β | P value | β | P value | OR | P value | OR | P value |
| rs28929474 | 14 | *SERPINA1* | T | -13.62 | 3.51E-08 | -0.08749 | 1.22E-08 | 2.312 | 7.82E-04 | 4.077 | 3.57E-03 |
| rs8079868 | 17 | *MYH3* | C | -6.669 | 5.93E-07 | -0.03441 | 3.50E-05 | 1.676 | 4.48E-05 | 1.501 | 3.80E-02 |
| rs4537555 | 1 | *HHAT* | G | -6.306 | 4.12E-06 | -0.04418 | 2.08E-07 | 1.272 | 5.23E-02 | 1.593 | 1.81E-02 |
| rs2165220 | 2 | *LPIN1* | C | -5.935 | 4.39E-06 | -0.02395 | 2.94E-03 | 1.302 | 2.41E-02 | 2.157 | 1.29E-04 |
| rs17016283 | 1 | *HHAT* | A | -6.777 | 4.59E-06 | -0.0435 | 2.23E-06 | 1.427 | 8.82E-03 | 1.824 | 5.06E-03 |
| rs12427132 | 12 | *SLCO1A2* | G | 8.328 | 5.44E-06 | 0.0428 | 1.75E-04 | 0.5875 | 1.31E-03 | 0.3581 | 4.52E-04 |
| rs6753618 | 2 | *GPR39* | A | 4.316 | 6.10E-06 | 0.02683 | 6.13E-06 | 0.7194 | 1.25E-04 | 0.6419 | 2.01E-03 |
| rs17167368 | 7 | *ETV1* | G | 15.55 | 7.66E-06 | 0.101 | 2.92E-06 | 0.2267 | 2.32E-05 | 0.4482 | 2.90E-01 |
| rs10489858 | 1 | *C1orf151* | C | -6.381 | 9.48E-06 | -0.03265 | 2.72E-04 | 1.522 | 1.75E-03 | 1.606 | 3.37E-02 |
| rs2281237 | 1 | *C1orf151* | T | -6.387 | 9.53E-06 | -0.03279 | 2.60E-04 | 1.514 | 2.03E-03 | 1.606 | 3.37E-02 |
| rs10503023 | 18 | *SEC11L3* | A | -4.581 | 9.57E-06 | -0.03109 | 1.34E-06 | 1.379 | 7.54E-04 | 1.392 | 2.67E-02 |
| rs6416788 | 16 | *CYLD* | A | -3.997 | 1.02E-05 | -0.01851 | 1.03E-03 | 1.353 | 2.29E-04 | 1.198 | 1.81E-01 |
| rs16956227 | 18 | *VAPA* | C | -9.359 | 1.24E-05 | -0.04732 | 3.84E-04 | 2.11 | 3.97E-04 | 2.206 | 2.07E-02 |
| rs17167977 | 7 | *DGKB* | T | -16.79 | 1.27E-05 | -0.08028 | 8.02E-04 | 2.292 | 3.26E-02 | 3.275 | 4.13E-02 |
| rs1888924 | 9 | *DAPK1* | G | 6.495 | 1.33E-05 | 0.03926 | 2.30E-05 | 0.6969 | 6.46E-03 | 0.5023 | 2.27E-03 |
| rs4775121 | 15 | *MYO1E* | A | 10 | 1.50E-05 | 0.04644 | 1.25E-03 | 0.6503 | 4.01E-02 | 0.3704 | 5.43E-03 |
| rs11076545 | 16 | *SALL1* | A | 3.925 | 1.60E-05 | 0.01323 | 1.96E-02 | 0.7705 | 1.60E-03 | 0.8167 | 1.32E-01 |
| rs2936582 | 5 | *ROPN1L* | A | -6.235 | 1.83E-05 | -0.03577 | 7.78E-05 | 1.63 | 4.17E-04 | 1.575 | 3.24E-02 |
| exm2255112 | 2 | *ECEL1* | A | -11.75 | 1.94E-05 | -0.06495 | 1.47E-04 | 2.477 | 1.36E-03 | 2.808 | 1.92E-02 |
| rs2962318 | 5 | *ROPN1L* | G | -6.184 | 2.02E-05 | -0.03597 | 6.74E-05 | 1.619 | 4.74E-04 | 1.552 | 3.67E-02 |
| rs12748886 | 1 | *HHAT* | G | 3.783 | 2.25E-05 | 0.02381 | 1.78E-05 | 0.8021 | 6.52E-03 | 0.6827 | 4.29E-03 |
| rs7585074 | 2 | *LPIN1* | G | -5.78 | 2.34E-05 | -0.02311 | 6.62E-03 | 1.271 | 5.22E-02 | 2.096 | 4.13E-04 |
| rs3000860 | 1 | *LOC12676* | T | -5.532 | 2.55E-05 | -0.03307 | 5.18E-05 | 1.443 | 2.60E-03 | 1.709 | 7.78E-03 |
| rs6766105 | 3 | *POLQ* | C | 4.055 | 2.74E-05 | 0.02086 | 5.28E-04 | 0.805 | 1.27E-02 | 0.6653 | 3.49E-03 |
| rs4741719 | 9 | *VLDLR* | C | -4.028 | 2.79E-05 | -0.02154 | 3.17E-04 | 1.315 | 1.76E-03 | 1.198 | 1.94E-01 |
| rs7514691 | 1 | *HHAT* | G | -3.666 | 3.09E-05 | -0.02465 | 6.58E-06 | 1.226 | 1.10E-02 | 1.489 | 2.47E-03 |
| rs4238819 | 16 | *CYLD* | G | 4.267 | 3.15E-05 | 0.02194 | 5.84E-04 | 0.7346 | 7.73E-04 | 0.7295 | 4.54E-02 |
| rs980249 | 9 | *SMC2L1* | G | -4.294 | 3.43E-05 | -0.01991 | 2.03E-03 | 1.391 | 4.74E-04 | 1.436 | 2.05E-02 |
| rs1419108 | 10 | *TCERG1L* | C | 3.753 | 3.49E-05 | 0.02035 | 3.09E-04 | 0.7701 | 1.23E-03 | 0.7618 | 4.13E-02 |
| rs9458989 | 6 | *QKI* | T | 6.209 | 3.87E-05 | 0.02225 | 1.79E-02 | 0.6559 | 2.22E-03 | 0.6284 | 2.98E-02 |
| rs4831399 | 8 | *DLC1* | C | -3.879 | 4.39E-05 | -0.02385 | 5.33E-05 | 1.433 | 4.36E-05 | 1.242 | 1.18E-01 |
| rs4782873 | 16 | *EFCBP2* | T | -3.632 | 4.54E-05 | -0.01915 | 5.50E-04 | 1.266 | 3.49E-03 | 1.2 | 1.64E-01 |
| rs4146 | 4 | *ADAM29* | A | -3.705 | 4.62E-05 | -0.02057 | 2.78E-04 | 1.316 | 8.52E-04 | 1.394 | 1.37E-02 |
| rs12612609 | 2 | *GPR39* | T | 5.237 | 4.93E-05 | 0.02891 | 3.15E-04 | 0.6737 | 7.34E-04 | 0.5144 | 5.48E-04 |
| rs199454 | 17 | *NSF* | G | -4.235 | 5.03E-05 | -0.01891 | 3.63E-03 | 1.281 | 8.75E-03 | 1.475 | 1.08E-02 |
| rs17472933 | 5 | *DAB2* | A | 3.572 | 5.19E-05 | 0.01933 | 4.32E-04 | 0.7862 | 2.34E-03 | 0.6599 | 2.21E-03 |
| rs6549723 | 3 | *CNTN3* | T | 8.729 | 5.23E-05 | 0.03826 | 4.41E-03 | 0.5747 | 5.30E-03 | 0.4558 | 1.74E-02 |
| rs11995187 | 8 | *LZTS1* | T | 4.496 | 5.57E-05 | 0.02472 | 3.68E-04 | 0.7755 | 1.10E-02 | 0.6961 | 3.22E-02 |
| rs130555 | 22 | *TIMP3* | C | -4.624 | 5.57E-05 | -0.02458 | 5.76E-04 | 1.555 | 4.35E-05 | 1.145 | 4.15E-01 |
| rs2357587 | 1 | *HHAT* | C | 3.534 | 5.63E-05 | 0.02397 | 1.10E-05 | 0.7216 | 4.19E-05 | 0.7739 | 4.61E-02 |
| rs2578283 | 9 | *GLDC* | T | 3.788 | 6.15E-05 | 0.01765 | 2.71E-03 | 0.7894 | 5.35E-03 | 0.7976 | 9.75E-02 |
| rs2087307 | 12 | *CLEC7A* | C | 3.913 | 6.27E-05 | 0.02177 | 3.44E-04 | 0.7683 | 2.97E-03 | 0.7621 | 5.54E-02 |
| rs1541123 | 6 | *EPHA7* | T | -3.734 | 6.34E-05 | -0.01724 | 3.01E-03 | 1.211 | 2.29E-02 | 1.458 | 6.61E-03 |
| rs8177822 | 9 | *ALAD* | A | 11.93 | 6.43E-05 | 0.05462 | 3.29E-03 | 0.5999 | 5.81E-02 | 0.2236 | 3.40E-03 |
| rs10929777 | 2 | *LPIN1* | C | -4.035 | 6.55E-05 | -0.01543 | 1.42E-02 | 1.125 | 1.94E-01 | 1.561 | 2.60E-03 |
| rs17400257 | 9 | *FRMD3* | A | 5.579 | 6.60E-05 | 0.033 | 1.48E-04 | 0.6984 | 3.85E-03 | 0.5569 | 7.05E-03 |
| rs875587 | 9 | *VLDLR* | G | -3.655 | 6.94E-05 | -0.02022 | 4.04E-04 | 1.273 | 3.47E-03 | 1.254 | 9.12E-02 |
| rs2567588 | 5 | *MARCH6* | G | -5.648 | 7.14E-05 | -0.03571 | 5.38E-05 | 1.539 | 1.22E-03 | 1.591 | 2.31E-02 |
| rs3816845 | 12 | *CLEC1A* | A | 5.205 | 7.36E-05 | 0.0281 | 5.81E-04 | 0.7121 | 4.25E-03 | 0.6879 | 4.60E-02 |
| rs9406600 | 9 | *BNC2* | T | 5.948 | 7.63E-05 | 0.03339 | 3.58E-04 | 0.7474 | 3.36E-02 | 0.44 | 3.30E-04 |
| rs862225 | 16 | *JPH3* | A | 6.611 | 7.69E-05 | 0.04879 | 2.63E-06 | 0.5239 | 1.73E-05 | 0.6117 | 5.23E-02 |
| rs10177527 | 2 | *GPR39* | C | 3.491 | 8.15E-05 | 0.02179 | 7.67E-05 | 0.7585 | 5.41E-04 | 0.7658 | 3.82E-02 |
| rs11689323 | 2 | *GPR39* | G | 3.491 | 8.15E-05 | 0.02179 | 7.67E-05 | 0.7585 | 5.41E-04 | 0.7658 | 3.82E-02 |
| rs2189132 | 22 | *MN1* | A | 3.66 | 8.28E-05 | 0.02146 | 2.06E-04 | 0.7835 | 3.53E-03 | 0.6205 | 5.21E-04 |
| rs448357 | 1 | *PTPRU* | A | 3.623 | 8.36E-05 | 0.01834 | 1.37E-03 | 0.7442 | 3.76E-04 | 0.6949 | 8.09E-03 |
| rs6815632 | 4 | *PCDH7* | G | -3.72 | 8.39E-05 | -0.01868 | 1.50E-03 | 1.337 | 6.47E-04 | 1.297 | 7.53E-02 |
| rs243957 | 4 | *ENPEP* | T | 3.762 | 8.46E-05 | 0.02381 | 6.29E-05 | 0.7376 | 3.71E-04 | 0.8329 | 1.91E-01 |
| rs17016349 | 1 | *HHAT* | A | -4.117 | 8.46E-05 | -0.0261 | 6.10E-05 | 1.174 | 8.77E-02 | 1.437 | 1.80E-02 |
| rs4600620 | 2 | *LPIN1* | A | -3.99 | 8.50E-05 | -0.01531 | 1.54E-02 | 1.108 | 2.62E-01 | 1.645 | 9.44E-04 |
| rs2988418 | 9 | *GLDC* | A | 4.356 | 8.57E-05 | 0.02051 | 2.97E-03 | 0.7638 | 7.36E-03 | 0.688 | 1.56E-02 |
| rs944546 | 9 | *VLDLR* | G | -3.611 | 8.72E-05 | -0.01883 | 1.01E-03 | 1.269 | 4.01E-03 | 1.238 | 1.10E-01 |
| rs4814858 | 20 | *SLC24A3* | C | -3.906 | 8.95E-05 | -0.02026 | 1.09E-03 | 1.287 | 5.83E-03 | 1.42 | 1.25E-02 |
| rs6064351 | 20 | *MC3R* | G | -4.288 | 8.96E-05 | -0.02487 | 2.60E-04 | 1.364 | 1.92E-03 | 1.438 | 2.28E-02 |
| rs10803742 | 2 | *LPIN1* | A | -3.99 | 9.32E-05 | -0.01479 | 2.00E-02 | 1.105 | 2.75E-01 | 1.641 | 1.09E-03 |
| rs1923786 | 9 | *FLJ45537* | A | 7.374 | 9.34E-05 | 0.04795 | 4.37E-05 | 0.5707 | 1.04E-03 | 0.4945 | 1.62E-02 |
| rs4365940 | 6 | *FUT9* | T | 3.921 | 9.41E-05 | 0.01938 | 1.92E-03 | 0.8132 | 2.34E-02 | 0.663 | 4.38E-03 |
| rs9485570 | 6 | *FUT9* | T | 3.937 | 9.88E-05 | 0.01991 | 1.54E-03 | 0.7934 | 1.15E-02 | 0.6504 | 3.17E-03 |
| rs17801458 | 2 | *LRP1B* | T | 7.382 | 9.93E-05 | 0.02439 | 3.89E-02 | 0.695 | 3.09E-02 | 0.7036 | 2.10E-01 |

*Top 10 SNPs (removing SNPs in strong LD: r^2^>0.8) used for joint analysis were colored in red.

Association analyses of Post-bronchodilator % predicted FEV_1_ and FEV_1_/FVC were performed using linear regression adjusted for age, sex, current smoking status, pack-years of cigarette smoking, and the first two principal components. Association analyses of COPD and COPD severity were performed using logistic regression adjusted for age, sex, current smoking status, pack-years of cigarette smoking, and the first two principal components.

**Table S3.** Genotype Frequency of rs28929474 in *SERPINA1* Stratified by GOLD Stages

|  | CC | CT | TT | MAF |
| --- | --- | --- | --- | --- |
| GOLD stage 0, n (%) | 539 (96.4) | 20 (3.58) | 0 (0) | 0.018 |
| GOLD stage 1, n (%) | 217 (97.7) | 5 (2.25) | 0 (0) | 0.011 |
| GOLD stage 2, n (%) | 472 (95.7) | 21 (4.26) | 0 (0) | 0.021 |
| GOLD stage 3, n (%) | 233 (88.3) | 25 (9.47) | 6 (2.27) | 0.070 |
| GOLD stage 4, n (%) | 98 (91.6) | 7 (6.54) | 2 (1.87) | 0.051 |
| All, n (%) | 1,559 (94.8) | 78 (4.74) | 8 (0.49) | 0.029 |

MAF: minor allele frequency of the T allele.

**Table S4.** Prediction Models for Post-bronchodilator Lung Function Using Top 10 SNPs for Post-bronchodilator % Predicted FEV_1_

|  | Post-bronchodilator FEV_1_/FVC | | | Post-bronchodilator % predicted FEV_1_ | | |
| --- | --- | --- | --- | --- | --- | --- |
|  | β | R^2^ | P value | β | R^2^ | P value |
| Genetic Score (4-13)* | -0.028 | 0.0749 | < 2.2 x 10^-16^ | -5.2 | 0.0991 | < 2.2 x 10^-16^ |
| Age at enrollment, years | -0.0024 | 0.0143 | 1.3 x 10^-6^ | -0.040 | 0.000160 | 0.61 |
| Sex (Male = 0, Female = 1) | 0.045 | 0.0190 | 2.3 x 10^-8^ | 1.86 | 0.00129 | 0.15 |
| Pack-years of cigarette smoking | -0.0011 | 0.0308 | 9.4 x 10^-13^ | -0.017 | 0.0296 | 2.5 x 10^-12^ |
| All | NA | 0.128 | < 2.2 x 10^-16^ | NA | 0.129 | < 2.2 x 10^-16^ |

*Genetic scores (the number of risk alleles) of the top 10 SNPs for post-bronchodilator % predicted FEV_1_ (rs28929474 in *SERPINA1*, rs8079868 in *MYH3*, rs4537555 in *HHAT*, rs2165220 in *LIPN1*, rs12427132 in *SLCO1A2*, rs6753618 in *GPR39*, rs17167368 in *ETV1*, rs10489858 in *C1orf151*, rs10503023 in *SEC11L3*, and rs6416788 in *CYLD*). 1,634 SPIROMICS non-Hispanic White smokers (GOLD stage 0-4) were included. Eight subjects with TT genotype of rs28929474 in *SERPINA1* (PiZZ genotype) were excluded.

**Figure S1.** Joint analysis of the top10 SNPs for post-bronchodilator % predicted FEV_1_ in 1,075 SPIROMICS non-Hispanic White smokers with COPD. Top 10 SNPs include rs28929474 in *SERPINA1*, rs8079868 in *MYH3*, rs4537555 in *HHAT*, rs2165220 in *LIPN1*, rs12427132 in *SLCO1A2*, rs6753618 in *GPR39*, rs17167368 in *ETV1*, rs10489858 in *C1orf151*, rs10503023 in *SEC11L3*, and rs6416788 in *CYLD*. Blue bars represent post-bronchodilator percent predicted FEV_1_, and red bars represent percentages of subjects with severe COPD (GOLD stage 3-4).


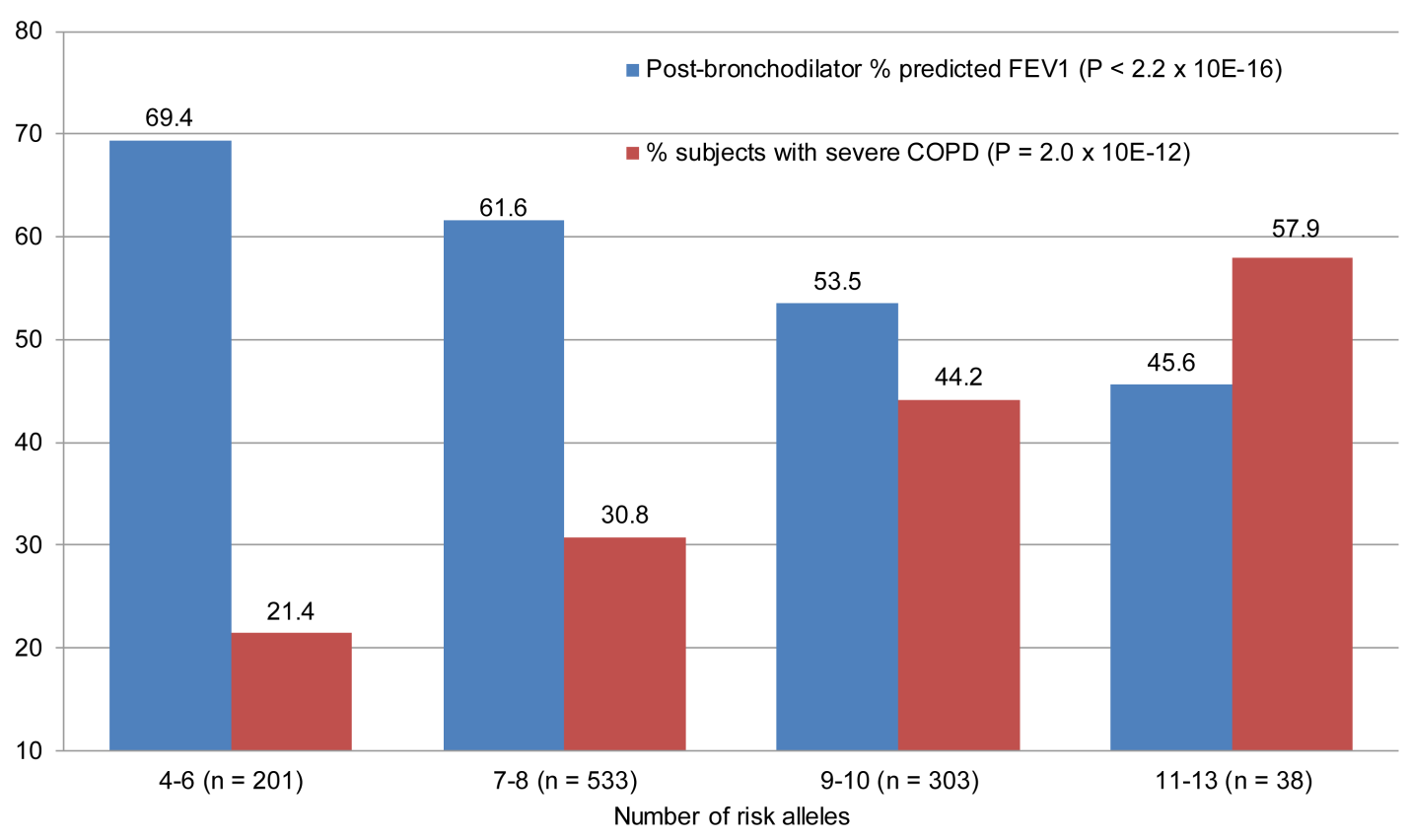

Supplement: Supplementary file 1 — Table S1. Association Results of the Top SNPs (P < 10− 4) with Post-bronchodilator FEV1/FVC. Table S2. Association Results of the Top SNPs (P < 10− 4) with Post-bronchodilator % Predicted FEV1.Table S3. Genotype Frequency of rs28929474 in SERPINA1 Stratified by GOLD Stages. Table S4. Prediction Models for Post-bronchodilator Lung Function Using Top 10 SNPs for Post-bronchodilator % Predicted FEV1.Figure S1. Joint analysis of the top10 SNPs for post-bronchodilator % predicted FEV1 in 1075 SPIROMICS non-Hispanic White smokers with COPD. (DOCX 141 kb) [file 12881_2018_656_MOESM1_ESM.docx]
